# Population pharmacokinetics of phenytoin after intravenous administration of fosphenytoin sodium in pediatric patients, adult patients, and healthy volunteers

**DOI:** 10.1007/s00228-012-1373-8

**Published:** 2012-08-24

**Authors:** Jun Tanaka, Hidefumi Kasai, Kenji Shimizu, Shigeki Shimasaki, Yuji Kumagai

**Affiliations:** 1 Clinical Study Management Division, Bell Medical Solutions Inc., Tokyu Bldg. East NO. 3, 2-16-8, Minami-Ikebukuro, Toshimaku, Tokyo, 171-0022 Japan; 2 Division of Research and Development, Nobelpharma Co. Ltd., Tokyo, Japan; 3 Clinical Trial Center, Kitasato University East Hospital, Kanagawa, Japan

**Keywords:** Fosphenytoin sodium injection, Phenytoin, Status epilepticus, Epileptic seizure, Population pharmacokinetics

## Abstract

**Purpose:**

We performed a population pharmacokinetic analysis of phenytoin after intravenous administration of fosphenytoin sodium in healthy, neurosurgical, and epileptic subjects, including pediatric patients, and determined the optimal dose and infusion rate for achieving the therapeutic range.

**Methods:**

We used pooled data obtained from two phase I studies and one phase III study performed in Japan. The population pharmacokinetic analysis was performed using NONMEM software. The optimal dose and infusion rate were determined using simulation results obtained using the final model. The therapeutic range for total plasma phenytoin concentration is 10–20 μg/mL.

**Results:**

We used a linear two-compartment model with conversion of fosphenytoin to phenytoin. Pharmacokinetic parameters of phenytoin, such as total clearance and central and peripheral volume of distribution were influenced by body weight. The dose simulations are as follows. In adult patients, the optimal dose and infusion rate of phenytoin for achieving the therapeutic range was 22.5 mg/kg and 3 mg/kg/min respectively. In pediatric patients, the total plasma concentration of phenytoin was within the therapeutic range for a shorter duration than that in adult patients at 22.5 mg/kg (3 mg/kg/min). However, many pediatric patients showed phenytoin concentration within the toxic range after administration of a dose of 30 mg/kg.

**Conclusions:**

The pharmacokinetics of phenytoin after intravenous administration of fosphenytoin sodium could be described using a linear two-compartment model. The administration of fosphenytoin sodium 22.5 mg/kg at an infusion rate of 3 mg/kg/min was optimal for achieving the desired plasma phenytoin concentration.

## Introduction

Fosphenytoin sodium was developed as a phenytoin prodrug to improve the low solubility of phenytoin sodium injection, which is used for the treatment of seizures, such as those in status epilepticus. Fosphenytoin sodium was approved in the United States in 1996 and subsequently in many other countries. Because the demand for fosphenytoin in Japan increased immediately after its approval in other countries, clinical trials were performed, and the drug was approved in July 2011.

Many studies have reported the population pharmacokinetics of phenytoin [1–3], but almost all studies have been performed at the steady-state trough levels of phenytoin. Ahn et al. [4] analyzed the population pharmacokinetics of phenytoin after fosphenytoin administration using plasma concentration data, including data from samples taken shortly after administration, but pediatric patients were not included in this study. We examined the population pharmacokinetics of phenytoin after intravenous administration of fosphenytoin sodium on the basis of data obtained from two phase I studies in healthy adult volunteers and one phase III study in pediatric and adult patients. Further, we established the optimal dose and rates of fosphenytoin sodium injection in Japan on the basis of these results.

## Materials and methods

### Study design

We conducted a population pharmacokinetic analysis of phenytoin by using pooled data obtained from two phase I studies and one phase III study. In addition, the data of phenytoin administration in phase I study was used in the population analysis.

In one of the phase I studies, 12 healthy adult volunteers participated in a single-dose, randomized, double-blind, two-period crossover study. Phenytoin sodium 250 mg and fosphenytoin sodium 375 mg were intravenously administered at infusion rates of 8.3 mg/min (administration time, 30 min) and 12.5 mg/min (30 min) respectively. The washout period between the two treatments was set at a minimum of two weeks.

In the second phase I study, 12 healthy adult volunteers participated in a single-dose, randomized, open-label, two-period, dose-escalation study. They were randomly allocated to one of two groups. Each group was intravenously administered a single dose of fosphenytoin sodium 563 mg at a rate of 18.8 mg/min (30 min) or 56.3 mg/min (10 min). After a two-week of washout period, fosphenytoin sodium 750 mg was intravenously administered at a rate of 25 mg/min (30 min) or 75 mg/min (10 min).

In phase I studies, 16 blood samples were collected from each subject before dosing, and at 10, 20, 30, 40, and 50 min, and 1, 1.25, 1.5, 2, 4, 8, 12, 24, 48, and 72 h after the start of drug administration.

In the phase III study, neurosurgical patients were intravenously administered fosphenytoin sodium 15 or 18 mg/kg at an infusion rate of 1 mg/kg/min (15 or 18 min) for the prevention of seizure after brain surgery or head trauma in an open-label manner. The epileptic patients, including those with status epilepticus and acute repetitive seizures, were intravenously administered fosphenytoin sodium 18 or 22.5 mg/kg at an infusion rate of 3 mg/kg/min (6 or 7.5 min for patients weighing <50 kg). The maximum infusion rate did not exceed 150 mg/min for any patient. Seven blood samples were collected from each patient before dosing and at 10 and 20 min, and 1, 2, 4, and 24 h after administration of drugs.

All studies were performed in accordance with the ICH Guidelines for Good Clinical Practice. The study protocols were reviewed and approved by the Institutional Review Boards of each clinical study site. Written informed consent was obtained from subjects or legally authorized representatives before participation in the study.

### Assay of total plasma phenytoin concentrations

Plasma samples were collected at each sampling point and were immediately frozen and stored at or below −20°C until analysis. Plasma samples from all studies were assayed at the time of each study in the Analysis Center (Sumika Chemical Analysis Service, Ltd., Osaka, Japan). Phenytoin was extracted from 20-μL plasma samples using diethyl ether under acidic conditions, and the phenytoin concentration was determined using liquid chromatography coupled with tandem mass spectrometry (LC/MS/MS). Dexamethasone sodium phosphate and mephenytoin were used as the internal standard. The linear range was obtained between 0.1 and 50 μg/mL (r ≥ 0.9976). Performance characteristics for LC/MS/MS assay are presented in Table 1. The quantitation method was validated for accuracy, precision, sensitivity, and specificity.

**Table 1:** Performance characteristics for liquid chromatography coupled with tandem mass spectrometry assay

Internal standard	Total phenytoin
	Mephenytoin
Linearity (μg/mL)	0.1–50 μg/mL (r ≥ 0.9974)
Interassay precision (%RSD)	3.6–6.4
Interassay accuracy (% relative error)	−10.1 to −3.4

### Population pharmacokinetic modeling

#### Software and algorithms

The population pharmacokinetic analysis of phenytoin was performed using the non-linear mixed effect model (NONMEM) software package, version VI level 2.0 [5]. The first order conditional estimation with interaction algorithm was used for parameter estimation. Conditional weighted residual (CWRES) was calculated based on the literature of Hooker et al. [6] using the R software [7]. Bootstrap resampling [8] was performed using the MULTTEST procedure of SAS software (SAS Institute Inc.).

#### Structural model

**Fig. 1:**
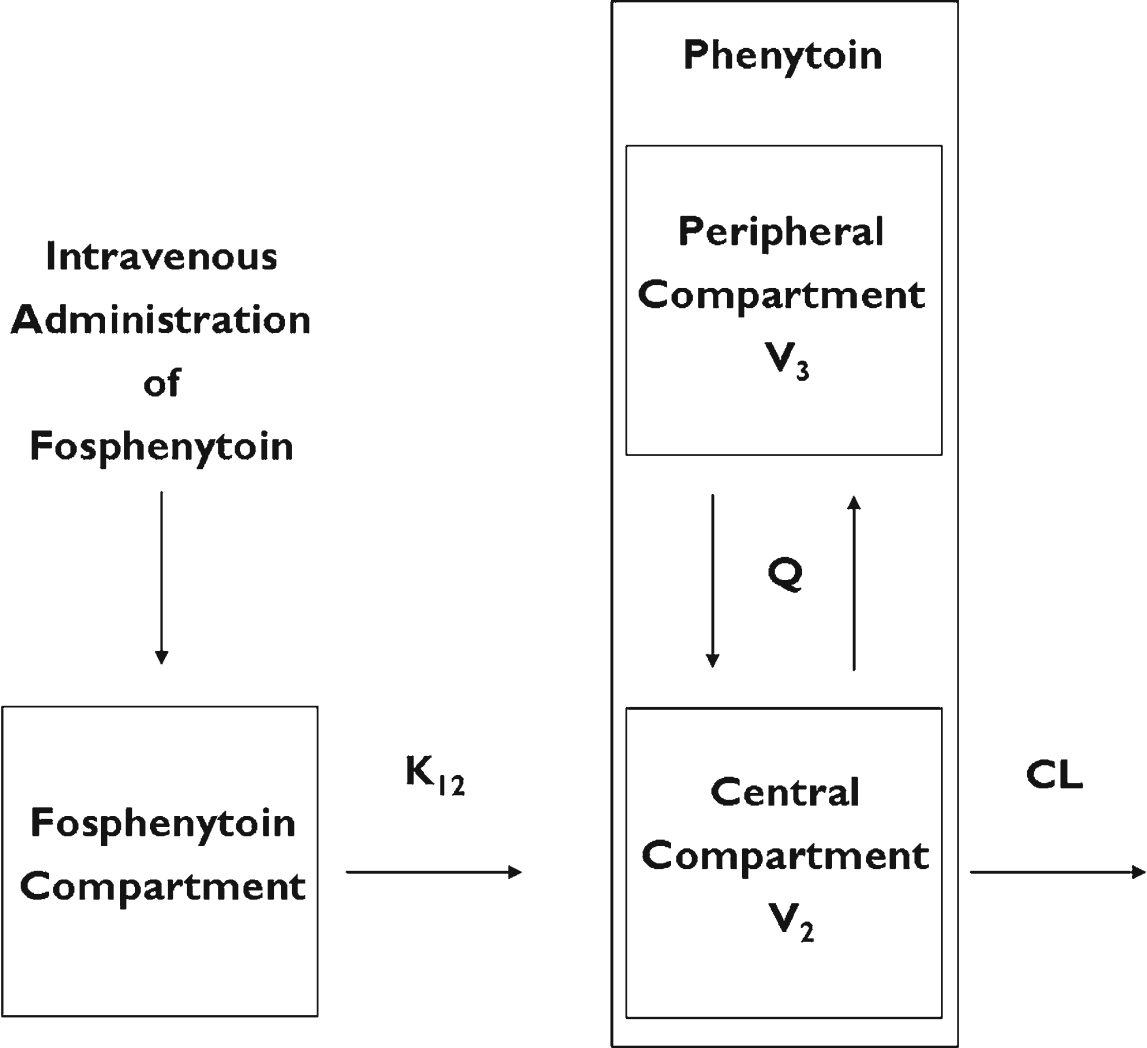
Structure of the two-compartment model with conversion of fosphenytoin to phenytoin

A linear two-compartment model with conversion of fosphenytoin to phenytoin (Fig. 1) was employed for the population pharmacokinetic analysis of phenytoin, because the maximum concentration (C_max_) of phenytoin indicated dose proportionality in the phase I study. The basic pharmacokinetic parameters were total clearance (CL, L/h); central volume of distribution (V_2_, L); inter-compartmental clearance (Q, L/h); peripheral volume of distribution (V_3_, L); and metabolism rate constant (K_12_, h^−1^). The bioavailability of phenytoin derived from intravenous fosphenytoin sodium injection is approximately 1 [9].

The inter-individual variability was investigated for all pharmacokinetic parameters. The inter-individual variability was calculated using an exponential error model as follows:

$$ {\theta _{ {\text{i}}}} = \theta \times {\text{exp}}\left( {{\eta _{ {\text{i}}}}} \right), $$

where θ_i_, is the i^th^ individual estimate of the parameter; θ, the typical population estimate; and η_i_, the normally distributed inter-individual random effect of mean 0 and variance ω^2^.

The residual variability was determined by using an exponential error model or combined exponential and additive error model as follows:

$$ {{\text{Y}}_{{{\text{ij}}}}} = {{\text{F}}_{{{\text{ij}}}}} \times {\text{exp}}\left( {{\varepsilon _{ {{\text{1ij}}}}}} \right) $$

$$ {{\text{Y}}_{{{\text{ij}}}}} = {{\text{F}}_{{{\text{ij}}}}} \times {\text{exp}}\left( {{\varepsilon _{ {\text{1}}}}_{{{\text{ij}}}}} \right) + {\varepsilon _{ {\text{2}}}}_{{{\text{ij}}}}, $$

where Y_ij_ and F_ij_ represent the j^th^ observed or predicted concentration for the i^th^ subject respectively, and ε_1ij_ and ε_2ij_ are the residual random effect of mean 0 and variance σ_1_^2^ and σ_2_^2^ respectively. The model judged to be the best structural model on the basis of the goodness-of-fit plots, 95% confidence interval of the parameter estimate, and the likelihood ratio test was chosen as the base model.

#### Covariate evaluation

Several covariates that could influence the pharmacokinetics of phenytoin were added one by one to the base model. The model that incorporated all possible covariates was chosen as the full model (forward step).

The effects of body weight (BW), age, gender, and trial patient type on each pharmacokinetic parameter were examined. For BW and age, a covariate model was developed using the power function model as follows:

$$ \theta = {\theta _{1}} \times {\left( {{{{{{{\text{COV}}}} \left/ {{{\text{COV}}}} \right.}}_{{{\text{typical}}\;{\text{value}}}}}} \right)^{{\theta {\text{COV}}}}}, $$

where θCOV is the influence factor to be estimated; θ_1_ the value of the pharmacokinetic parameter for subject with typical value of covariate; and θ, the typical value of the pharmacokinetic parameter.

Gender and trial patient type were modeled as follows:

$$ \theta = {\theta _{1}} \times {\left( {{\theta _{ {{\text{cov}}}}}} \right)^{{{\text{cov}}}}}, $$

where the variable cov was assigned a value of 0 for men or non-applicable subjects and 1 for women or applicable subjects and θ_cov_ was a covariate difference in θ.

After the forward step, each covariate in the full model was tested in turn by removing each entity one by one to confirm the statistical significance. The model with only significant covariates was chosen as the final model (backward elimination step).

#### Model evaluation

The population pharmacokinetic model was evaluated on the basis of goodness-of-fit plots, 95% confidence interval for the parameter estimates, and the likelihood ratio test. The 95% confidence interval for parameter estimates was obtained from the point estimate ± 1.96 × standard error (SE), which was taken from the covariance step. The coefficient of variance (CV [%]) for individual variability and intra-individual variability were calculated from the square root of the variance. In the likelihood ratio test, the objective function value difference (ΔOBJ) was used for evaluating the statistical significance of the parameters. The *p* values for the forward selection step and backward elimination step were <0.05 (ΔOBJ was < 3.84) and *P* < 0.01 (ΔOBJ was > 6.63) respectively.

Bootstrap validation was used to evaluate the validity and robustness of the final model. Two hundred data sets were reconstructed by resampling the subjects from the original data set. Successful estimation was defined as the normal completion of both the estimation and covariance steps of the NONMEM software. The mean parameter estimates and standard error obtained from the bootstrap replications were compared with the final parameter estimates and standard error obtained from the original data set. The model for which the probability of a successful bootstrap run was more than 90% and the parameter estimates were comparable was defined as the robust model.

#### Simulation

The optimal dose and infusion rate were examined on the basis of the results of the simulation. The simulations of pediatric and adult patients using the final population model were produced using data sets of 200 subjects depending on the following doses and rates. The doses and rates used for patients in the study and the maximum dose in the United States (30 mg/kg) were selected. The data sets for pediatric and adult patients were reconstructed by resampling the values from the covariate data observed in the phase III study. The simulations were performed to check for profiles that remained within the therapeutic range and those that crossed the toxicity threshold:

1. Dose of 15 mg/kg body weight (rate: 1 mg/kg/min)
2. Dose of 18 mg/kg body weight (rate: 1 mg/kg/min)
3. Dose of 18 mg/kg body weight (rate: 3 mg/kg/min)
4. Dose of 22.5 mg/kg body weight (rate: 3 mg/kg/min)
5. Dose of 30 mg/kg body weight (rate: 3 mg/kg/min)

## Results

**Fig. 2:**
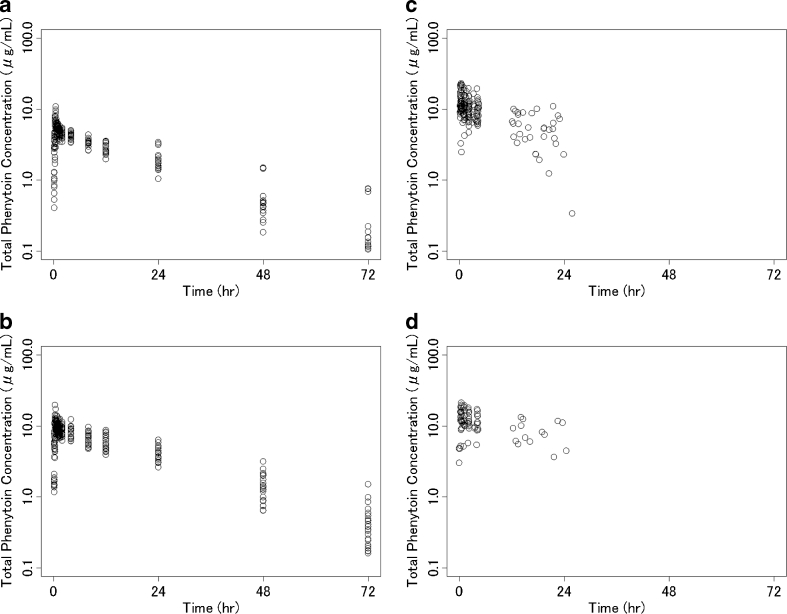
Total plasma phenytoin concentrations obtained from clinical studies. **a** Healthy volunteers (*n* = 12) in a Phase I study (study in which phenytoin sodium and fosphenytoin sodium were administered). **b** Healthy volunteers (*n* = 12) in a Phase I study (dose-escalation study). **c** Pediatric patients (*n* = 33) in a Phase III study. **d** Adult patients (*n* = 14) in a Phase III study

The population pharmacokinetic analysis was performed using data 923 plasma concentrations collected from 24 healthy volunteers, 14 adult patients, and 33 pediatric patients. The plot of time versus total plasma phenytoin concentration is shown in Fig. 2. Descriptive statistics are presented in Table 2.

**Table 2:** Summary of patients used in the evaluation

Demographic	All patients	Phase I study patients	Phase III study patients (adult patients [ ≥ 17])	Phase III study patients (pediatric patients [2–16])
Gender (man/woman)	44/27	24/0	7/7	13/20
Age (years)^a^	19.6 ± 15.6 (2–86)	24.9 ± 5.1 (20–37)	38.2 ± 21.1 (17–86)	7.8 ± 4.3 (2–16)
Body weight (kg)^a^	43.8 ± 21.3 (7.8–74.4)	64.8 ± 5.1 (56.9–74.4)	53.5 ± 9.9 (39.0–72.2)	24.4 ± 13.0 (7.8–60.3)

^a^Arithmetic mean ± standard deviation (minimum to maximum)

The pharmacokinetics of phenytoin was well-described using the two-compartment model with conversion of fosphenytoin to phenytoin, which included the inter-individual variability of all pharmacokinetic parameters. Residual variability was modeled using a combined error. In the forward selection, CL, V_2_, and V_3_ were influenced by BW. In addition, V_3_ was influenced by age and gender, but the influence of these parameters was not statistically significant. Therefore, V_3_ was related only to BW. The average BW of adult Japanese men (60 kg) was selected as the standard value. In the backward elimination, no covariate was eliminated from the full model. The influence factor of V_2_ was fixed to 1 on the basis of statistical significance (*P* < 0.01). The final model and its parameter estimates are shown in Table 3 and Eq. 1.

**Table 3:** Parameter estimates from the final population pharmacokinetic model and results of the bootstrap analysis

Parameter		Estimate	Standard error	95% CI	Bootstrap estimate	Bootstrap standard error	Bootstrap 95% CI
Population mean							
CL (L/h)^a^	θ_1_	1.61	0.0933	1.43–1.79	1.61	0.0878	1.48–1.74
Θ_WT_^(CL)^	θ_2_	0.569	0.0862	0.400–0.738	0.575	0.0912	0.431–0.734
V_2_ (L)^a^	θ_3_	20.8	1.99	16.9–24.7	20.3	2.68	16.1–25.0
Q (L/h)	θ_4_	53.0	4.75	43.7–62.3	53.4	5.87	44.9–64.1
V_3_ (L)^a^	θ_5_	26.0	1.55	23.0–29.0	26.5	2.43	22.8–30.7
Θ_WT_^(V3)^	θ_6_	0.584	0.0434	0.499–0.669	0.591	0.0520	0.495–0.674
K_12_ (1/h)	θ_7_	5.02	0.399	4.24–5.80	4.96	0.518	4.21–6.00
Inter-subject variability							
ω_CL,CL_	ω_1,1_	0.194	0.0391	0.117–0.271	0.190	0.0348	0.135–0.252
ω_V2,V2_	ω_2,2_	0.161	0.0576	0.0481–0.274	0.133	0.0684	0.0164–0.236
ω_Q,Q_	ω_3,3_	0.271	0.0840	0.106–0.436	0.289	0.119	0.121–0.494
ω_V3,V3_	ω_4,4_	0.0430	0.0167	0.0103–0.0757	0.0470	0.0214	0.0179–0.0900
ω_K12,K12_	ω_5,5_	0.106	0.0483	0.0110–0.201	0.112	0.0565	0.0377–0.209
Intra-individual variability							
σ^2^ (exponential error)	σ_1,1_	0.00148	0.000692	0.000124–0.00284	0.00181	0.00110	0.000495–0.00343
σ^2^ (additive error)	σ_2,2_	0.317	0.0977	0.126–0.508	0.304	0.100	0.159–0.473

^a^CL, V2, and V3 are typical values for an individual weighing 60 kg

1 $$ \matrix{  {{\text{CL}} = {\theta _{ {{\text{CL}}}}}*{{\left( {{{{{\text{WT}}}} \left/ {{{\text{6}}0}} \right.}} \right)}^{{\theta {\text{wt}}\left( {{\text{CL}}} \right)}}}*{{\text{e}}^{{\eta {\text{CL}}}}}} \hfill \\ {{{\text{V}}_{{\text{2}}}} = {\theta _{ {{\text{V2}}}}}*\left( {{{{{\text{WT}}}} \left/ {{{\text{6}}0}} \right.}} \right)*{{\text{e}}^{{\eta {\text{V2}}}}}} \hfill \\ {{\text{Q}} = {\theta _{ {\text{Q}}}}*{{\text{e}}^{{\eta {\text{Q}}}}}} \hfill \\ {{{\text{V}}_{{\text{3}}}} = {\theta _{ {V3}}}*{{\left( {{{{{\text{WT}}}} \left/ {{{\text{6}}0}} \right.}} \right)}^{{\theta {\text{wt}}\left( {{\text{V3}}} \right)}}}*{{\text{e}}^{{\eta {\text{V3}}}}}} \hfill \\ {{{\text{K}}_{{{\text{12}}}}} = {\theta _{ {{\text{K12}}}}} * {{\text{e}}^{{\eta {\text{K12}}}}}} \hfill \\ }<!end array> $$

**Fig. 3:**
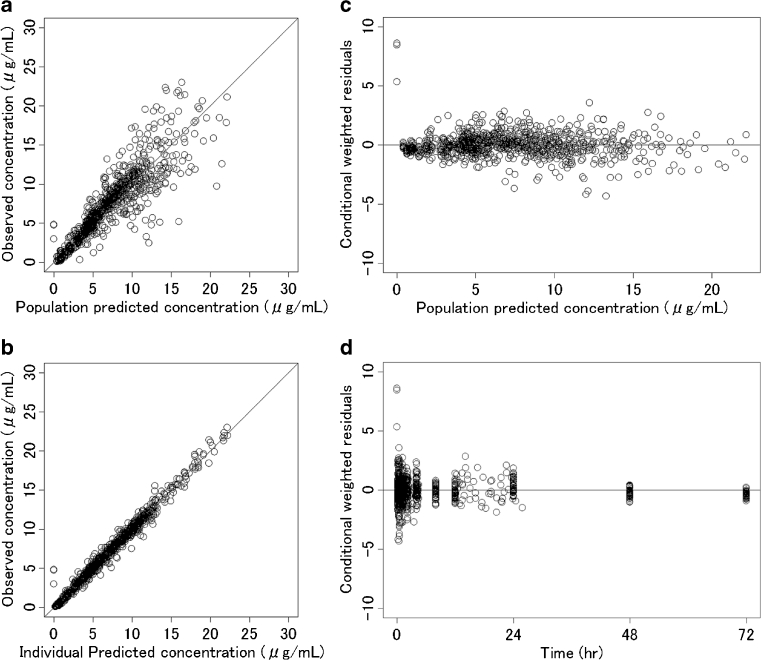
Goodness-of-fit plots for the final population pharmacokinetic model for fosphenytoin. **a** The population-predicted concentrations versus the observed concentrations. **b** The individual predicted concentrations versus the observed concentrations. **c** The population-predicted concentrations versus the individual conditional weighted residuals. **d** Time versus the individual conditional weighted residuals

The goodness-of-fit plots of the final model are shown in Fig. 3. The concentrations predicted using the model and the individual predicted concentrations were consistent with the observed concentrations. The plots for time and the model predicted concentration versus CWRES presented good distribution around 0.

The results of the bootstrap validation are shown in Table 3. The success rate was 91.0%, and the estimated population values and the average of the bootstrap results were fairly consistent, which suggested good stability in parameter estimates.

**Fig. 4:**
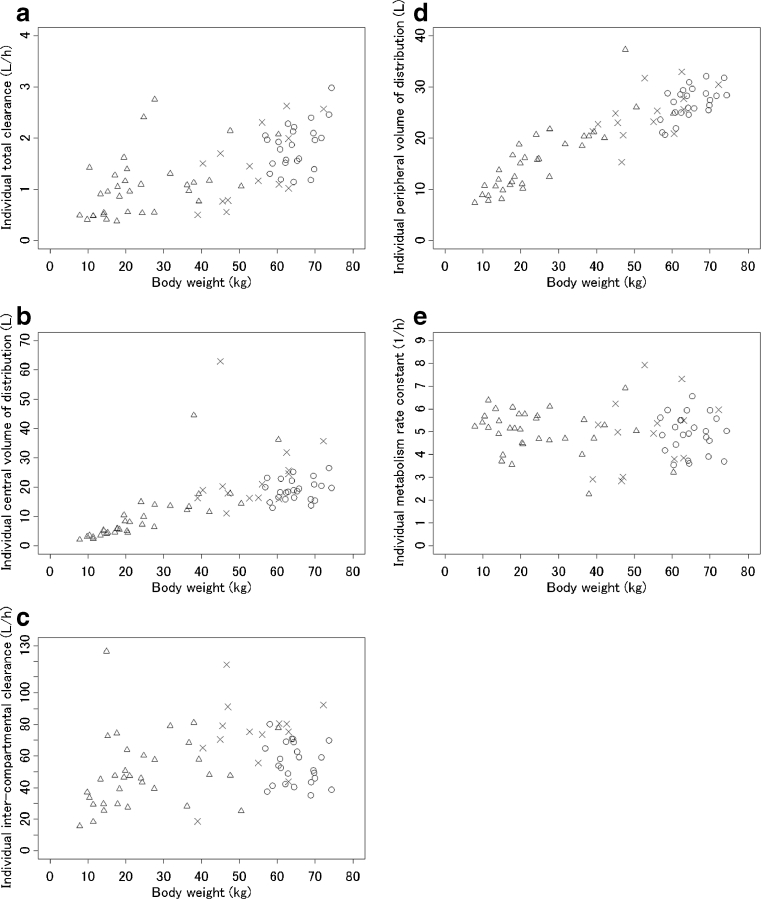
The individual pharmacokinetic parameters of the subjects based on the final model. *Triangle*, pediatric patients (*n* = 33); *cross*, adult patients (*n* = 14); *circle*, healthy volunteers (*n* = 24)

The individual pharmacokinetic parameters of the subjects based on the final model are shown in Fig. 4.

**Fig. 5:**
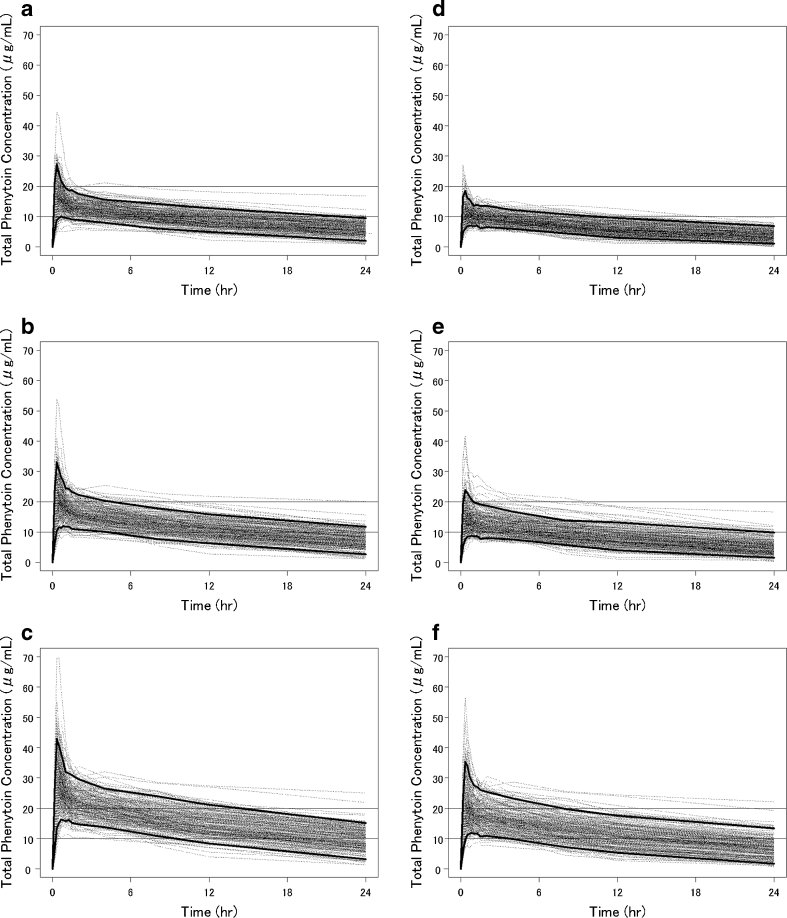
The simulated total plasma phenytoin concentrations. **a** Adults, dose: 18 mg/kg. **b** Adults, dose: 22.5 mg/kg. **c** Adults, dose: 30 mg/kg. **d** Children, dose: 18 mg/kg. **e** Children, dose: 22.5 mg/kg. **f** Children, dose: 30 mg/kg; rate, 3 mg/kg/min). *Dotted line*, individual simulated concentrations using the final model; *solid line*, 95 percentile or 5 percentile of predicted values using the final model

The dose simulation for determining the optimal dose and rate of infusion for pediatric patients and adult patients are as follows (Fig. 5): in adult patients, the C_max_ of total plasma phenytoin was within the therapeutic range (10–20 μg/mL) at doses of 18 mg/kg (rates of 1 and 3 mg/kg/min) or less, but the drug concentration did not remain over 10 μg/mL for a long duration. The appropriate profile for retaining the therapeutic range was shown at a dose of 22.5 mg/kg with a rate of 3 mg/kg/min. On the other hand, C_max_ values were more than 20 μg/mL in almost all simulations at a dose of 30 mg/kg. In pediatric patients, the total plasma phenytoin concentration remained within the therapeutic range from 10 to 20 μg/mL for a short duration at a dose of 18 mg/kg or less. The concentration ranged from 10 to 20 μg/mL for shorter duration in pediatric patients than in adult patients at 22.5 mg/kg (a rate of 3 mg/kg/min). Many pediatric patients showed phenytoin concentration in the toxic range when administered at a dose of 30 mg/kg.

## Discussion

Although our study was a retrospective population pharmacokinetic analysis, all the data obtained from the studies conducted in Japan were used.

Phenytoin metabolism can be saturated if sufficiently high doses are administered [10], but the C_max_ values showed the dose proportionality in this dose escalation study. Therefore, the saturation of the metabolism of phenytoin was not a problem in this study, and the pharmacokinetics of phenytoin after intravenous administration of fosphenytoin sodium was described using a linear two-compartment model.

**Fig. 6:**
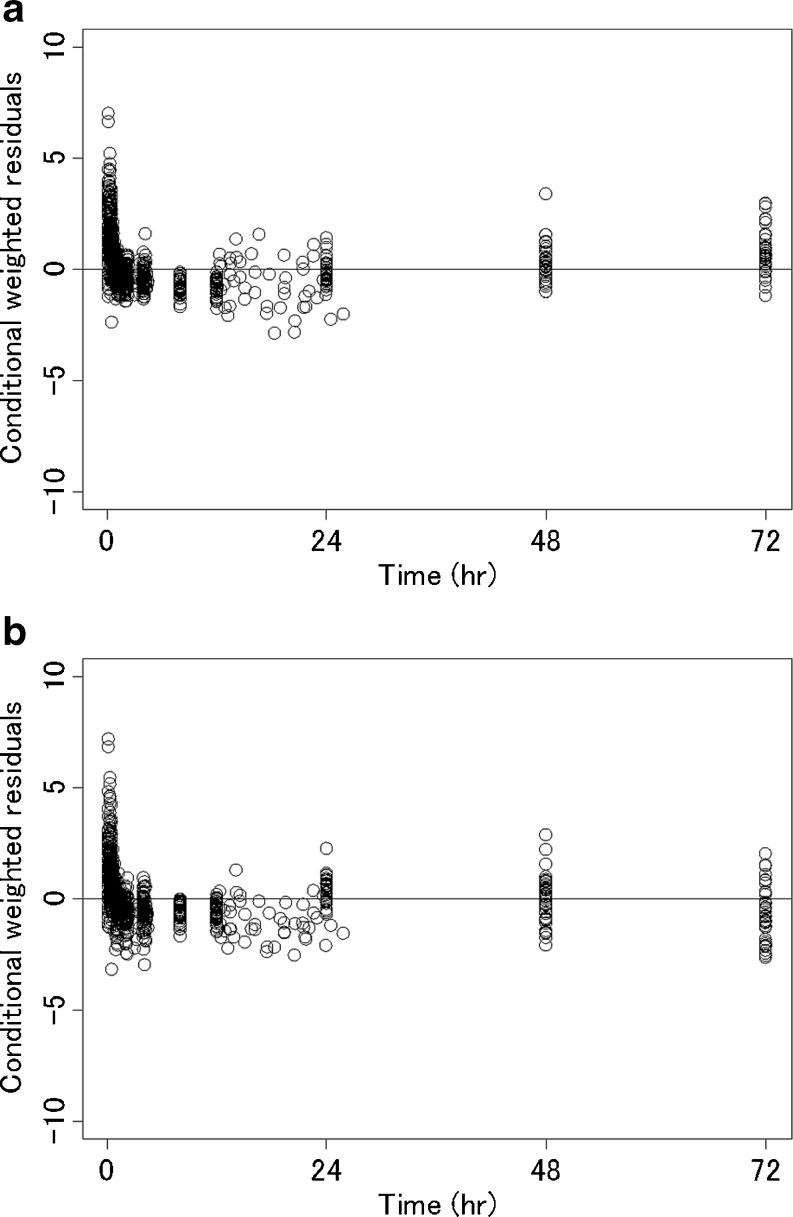
Time versus the individual conditional weighted residuals plots using our data. **a** Odani’s population pharmacokinetic model. **b** Ahn’s population pharmacokinetic model

The residual plots of data obtained using Odani’s and Ahn’s population pharmacokinetic models are shown in Fig. 6 [1, 4]. Odani’s model was a one-compartment model with Michaelis–Menten (MM) elimination. Maximal elimination rate (V_max_) and volume of distribution (Vd) were adjusted for hypothetical body size. Ahn used a linear one-compartment model with drug depot compartment for fosphenytoin. CL and Vd were adjusted for body weight. Plots for time versus CWRES reflected a bias from 0 to 24 h in both the models. Neither of the one-compartment models could predict the total plasma phenytoin concentrations in the distribution phase. Therefore, the one-compartment model was inappropriate for studying the pharmacokinetics of phenytoin after intravenous fosphenytoin sodium injection. All pharmacokinetic parameters correlated with body weight in the present final model and in Odani’s model and Ahn’s model. The Vd of the present final model was calculated from V_2_ and V_3_. The CL was about 1.4 times higher and the Vd was about 1.5 times higher at all body weights in Odani’s model. It was difficult to compare our final model against Odani’s model, because the structural model between these two models was different. However, with respect to Vd, the informative plasma samples that were collected immediately after injection were higher in the present study. Therefore, the estimated Vd in this study was considered more plausible. The Vd estimates obtained using the final model and Ahn’s model were nearly the same, but the CL of Ahn’s model was about 0.6 times lower at all body weights. This was because Ahn’s model was built on the basis of steady-state data and the data of elderly subjects.

The estimated half-life of conversion from fosphenytoin to phenytoin calculated from K_12_, which was approximately 8 min, was consistent with the half-life proposed by Boucher et al. [9].

In this study, we used total phenytoin concentration. Therefore, the ramification that fosphenytoin displaces phenytoin from albumin had limited effects.

We used a limited number of patients taking medications, which may have affected the pharmacokinetics. Therefore, we could not examine the influence of concomitant medications.

Many studies have reported that genetic polymorphisms in the drug metabolizing enzymes CYP2C9 and 2C19 can greatly affect the pharmacokinetic parameters of phenytoin in Japanese populations [11–13]. However, genetic information was not collected in this study. Further, collection of this information during routine clinical practice would be difficult. Therefore, we did not examine the influence of genetic polymorphisms in this analysis.

The therapeutic range for phenytoin is considered to be 10–20 μg/mL as the total plasma phenytoin concentrations in patients with normal plasma protein binding [10]. Therefore, we used this index for the simulations.

The results of the simulations showed that the total plasma phenytoin concentrations in adult patients were higher than those in pediatric patients. However, administration of 22.5 mg/kg of fosphenytoin sodium at an infusion rate of 3 mg/kg/min was considered to lead to an appropriate profile of total plasma phenytoin concentration in the two groups.

In this study, plasma samples were collected from subjects after single-dose administration. Therefore, we performed only single-dose simulation and proposed a single-dose regimen. Further, administration of a maintenance dose is more effective in clinical practice.

Our proposed dose regimen was higher than the approved dose cited in the package insert for phenytoin injection in Japan. However, differences in formulation will have more clinical implications. Therefore, differences in the dose regimen would have almost no impact.

## Conclusion

A linear two-compartment model was effective for population pharmacokinetic analysis of phenytoin after intravenous administration of fosphenytoin sodium because no bias was observed in the goodness-of-fit plots obtained using the final model. Therefore, we established the optimal dosage of fosphenytoin sodium injection on the basis of this model. The model and optimal dosage in clinical practice require verification.
